# Study on the Tensile Behavior of Woven Non-Woven PLA/OLA/MgO Electrospun Fibers

**DOI:** 10.3390/polym15193973

**Published:** 2023-10-03

**Authors:** Adrián Leonés, Laura Peponi, Jesús-María García-Martínez, Emilia P. Collar

**Affiliations:** Instituto de Ciencia y Tecnología de Polímeros (ICTP-CSIC), Juan de la Cierva 3, 28006 Madrid, Spain; aleones@ictp.csic.es (A.L.); jesus.maria@ictp.csic.es (J.-M.G.-M.); ecollar@ictp.csic.es (E.P.C.)

**Keywords:** electrospinning, poly(lactic acid), magnesium oxide, oligomer(lactic acid), design of experiments, Box–Wilson response surface methodology

## Abstract

The present work deeply studied the mechanical behavior of woven non-woven PLA/OLA/MgO electrospun fibers, efibers, by using Box–Wilson surface response methodology. This work follows up a previous one where both the diameters and the thermal response of such efibers were discussed in terms of both the different amounts of magnesium oxide nanoparticles, MgO, as well as of the oligomer (lactic acid), OLA, used as plasticizer. The results of both works, in term of diameters, degree of crystallinity, and mechanical response, can be strongly correlated to each other, as reported here. In particular, the strain mechanism of PLA/OLA/MgO efibers was studied, showing an orientation of efibers parallel to the applied stress and identifying the mechanically weakest points that yielded the start of the breakage of efibers. Moreover, we identified 1.5 wt% as the critical amount of MgO, above which the plasticizing effect of OLA was weaker as the amount of both components increased. Moreover, the minimum elastic modulus value took place at 15 wt% of OLA, in agreement with the previously reported convergence point in the evolution of the degree of crystallinity. Regarding the yield point, a concentration of OLA between 20 and 30 wt% led to a slight improvement in the yielding capability in terms of tensile strength in comparison with neat PLA efibers. Therefore, the approach presented here permits the design of tailor-made electrospun nanocomposites with specific mechanical requirements.

## 1. Introduction

Nowadays, poly(lactic acid), PLA, has emerged as a prominent polymer substitute for various petrochemical-based polymers due to its remarkable properties, such as biodegradability [1,2] or compostability [3,4]. However, from the mechanical point of view, its inherent brittleness and poor elongation at break has limited its applications [5]. This limitation is particularly significant in fields such as packaging [6,7] or tissues engineering [8]. Certainly, the brittleness of PLA constitutes a major problem in the main industrial fields, where there is no tolerance for cracking or tearing when subjected to force during manufacturing [9,10].

Efforts have been carried out to enhance these mechanical properties, including blending with other biodegradable polymers [11,12], copolymerization with other polymers [13,14], reinforcing with nanoparticles, NPs [15,16], or the incorporation of plasticizers agents [17,18,19,20]. Particularly, the design of PLA-based nanocomposites by adding NPs represents one of the most used strategies to increase and to enhance the mechanical properties of PLA [12,21].

Regarding the different processing techniques used to obtain PLA-based nanocomposites, the electrospinning technique has emerged as a suitable technique to fabricate PLA-based electrospun nanocomposites. This versatile technique enables the easy fabrication of woven non-woven electrospun fibers (from now on, referred to as efibers) reinforced with NPs through the application of a high electric voltage to a dispersion of NPs within a polymer solution in a volatile solvent under ambient conditions [8,22]. In particular, for biomedical applications, searching to improve the mechanical properties of pristine PLA efibers to mimic those of human tissues, the use of organic NPs is reported in the scientific literature, such as cellulose nanocrystals, CNC, or chitosan NPs with enhanced elongation at break and higher degree of crystallinity [23]. On the other hand, also, inorganic NPs such as magnesium oxide, MgO, NPs or silver, Ag, NPs can be used as reinforcements for PLA-based efibers due to their biocompatibility and antimicrobial properties [8,24,25].

Therefore, electrospun PLA-based nanocomposites represent the combination of, at least, two different components—in particular, the combination of the PLA polymeric matrix with other materials on the nanometric scale, such as NPs, in order to significantly improve their mechanical properties [12]. The improvement in the mechanical properties of efibers is related to the existence of interactions between NP surfaces and the macromolecular chains of the PLA matrix that surround them. Specifically, these interactions take place at the molecular level, where physical–chemical interactions between NPs and PLA chains play a crucial role in the overall behavior of the material as a whole [26]. Such a level of molecular interactions take place at the nanoscale and are related to the mobility of polymer chains adjacent to NPs and can be affected by some factors, such as the length of the polymer chains [27,28], the NP surface morphology [29,30], the NP concentration [31], or the interactions between polymer NPs and other components [32].

Furthermore, to properly tailor the thermal properties of electrospun PLA-based nanocomposites, such as its glass transition temperature, T_g_, to approach the human body, the incorporation of plasticizers has been studied in the scientific literature [17,18]. The ideal plasticizer should exhibit a similar chemical structure, showing comparable intermolecular forces capable of strongly interacting with the PLA matrix. Additionally, oligomeric lactic acid, OLA, presents itself as a promising alternative to conventional plasticizers for PLA due to its similar chemical structure, relatively high molar mass, and renewable origin [33,34,35]. In fact, looking at the increase of both the mechanical and the thermal properties, preliminary studies of PLA-based nanocomposites plasticized with OLA can be found in the literature [17,36]. From both the mechanical and thermal points of view, Arrieta et al. reported a significant increase of the elongation at break of 20% with 15 wt% of OLA in comparison with neat PLA-based efibers [36], and Leonés et al. reported an increase in the elongation at break with 10–30 wt% OLA content, along with effective modulation of the glass transition temperature of PLA-based efibers [17].

Additionally, a certain concentration of plasticizer will be considered as the maximum, named the critical concentration [37], and, consequently, different amounts of plasticizer are expected to cause increasing improvements of the molecular interactions between PLA chains and NPs up to a limit close to the critical concentration. Once this critical concentration is overpassed, no further enhancements in the molecular interactions will occur between the components. In fact, above this concentration, the structural integrity of the polymer matrix could be compromised, leading to the possible segregation of the plasticizer, which could form its own phase decreasing the thermal and mechanical properties of the nanocomposites [38].

Therefore, understanding the interactions between NPs, plasticizer, and the surrounding polymeric chains implies an important aspect to be considered in the study of nanocomposites and their final mechanical performance. In this work, we describe the fitting of the mechanical behavior of PLA/OLA/MgO electrospun fibers to a mathematical model that permits the design of tailor-made electrospun nanocomposites with specific mechanical requirements. With this aim, and based on our previous results [18], the purpose of the present study is to find a correlation between each one of the tensile parameters and the composition of the PLA/OLA/MgO electrospun PLA-based nanocomposites. Thus, a Box–Wilson surface response methodology statistical design was used to model the system behavior in all the experimental range scanned. Once the PLA-based efibers were obtained, tensile tests were carried out, and the main mechanical properties were calculated. From these, a series of remarks concerning the tensile mechanism between MgO NPs, OLA plasticizer, and the PLA matrix was proposed and properly discussed in this work.

## 2. Materials and Methods

Polylactic acid (PLA3051D, 3% of D-lactic acid monomer, molecular weight 14.2 × 10^4^ g·mol^−1^, density 1.24 g·cm^−3^) was supplied by NatureWorks^®^, Minneapolis, MN, USA. Lactic acid oligomer (Glyplast OLA8, ester content >99%, density 1.11 g·cm^−3^, viscosity 22.5 mPa·s, molecular weight 1100 g·mol^−1^) was kindly supplied by Condensia Quimica SA, Barcelona, Spain.

Before starting the electrospinning process, each solution was prepared following the next process. Firstly, the corresponding amounts of PLA and OLA were dissolved separately in CHCl_3_ and stirred overnight at room temperature. Secondly, the amount of MgO NPs was weighed and dispersed in 20 mL of CHCl_3_; after 30 min, the OLA solution was added to the MgO NPs solution and dispersed over 60 min. Then, the PLA solution was added to the MgO NPs and OLA solution and dispersed simultaneously for another 60 min. Finally, the necessary volume of DMF was added to assure the proportion of solvents (CHCl_3_:DMF, 4:1). The dispersion process was carried out with a sonicator tip (Sonic Vibra-Cell VCX 750) of 750 watts and amplitude of 20%. Then, electrospun fiber mats were obtained in an Electrospinner Y-flow 2.2.D-XXX (Nanotechnology Solutions, Malaga, Spain). The electrospinning parameters were set as a voltage of 20 kV, distance of 17 cm between the tip and collector, and a polymer solution flux of 3.5 mL·h^−1^ in order to obtain randomly oriented efiber mats [18].

The amounts of OLA (wt%) and MgO (wt%) were determined in agreement with the specifications of the Box–Wilson experimental worksheet (Table 1). Box–Wilson methodology is based on a factorial design involving 2*k* + 2*k* + 1 experiments with 2 + *K* additional replicated central runs, where *k* represents the number of independent variables [39,40]. The central point variables are coded as (0, 0), while the independent variable interval (OLA and MgO) is in the range between 6.00 and 30.00 wt% for OLA and between 0.60 and 3.00 wt% for MgO due to the factorial component being coded as (−1, 1). Additionally, the Box–Wilson experimental model considers α = √2 as the coded variable for the star points of the worksheet [39,40]. Therefore, all coded and controlled factors are listed in Table 1 together with each run number. The trials were carried out in a randomized way, and the variables studied were statistically analyzed by one-way analysis of variance (ANOVA) using the statistical computer package Statgraphics Centurion XVII (Statpoint Technologies, Inc., Warrenton, VA, USA) [18].

Once the different polynomials fitted to quadratic models of each mechanical property studied by following the Box–Wilson surface response methodology were obtained, the data were then plotted using the 3D response surface plot, as well as the contour plots. In particular, the polynomial models corresponding to each mechanical property were plotted as a function of the OLA and MgO NP contents in all the experimental range scanned using OriginPro v8.5 software.

Once the efibers mat of each experimental run was obtained, the morphological and mechanical characterization was carried out. Scanning Electron Microscopy, SEM (PHILIPS XL30 Scanning Electron Microscope, Phillips, Eindhoven, The Netherlands), and Field Emission Scanning Electron Microscopy, FESEM (Hitachi S8000), were used in order to study the morphology of the efibers. All the samples were previously gold-coated (~5 nm thickness) in a Polaron SC7640 Auto/Manual Sputter (Polaron, Newhaven, East Sussex, UK).

The mechanical properties of the PLA-based electrospun nanocomposite mats were studied in the tensile test mode at room temperature. For this purpose, an Instron dynamometer (model 3366) equipped with a 100 N load cell at a crosshead speed of 10 mm·min^−1^ and initial length between clamps of 30 mm was used. At least five specimens of 10 mm, 6 mm width, and 100 µm of average thickness cut from the electrospun mats were measured. The mechanical properties identified over the stress–strain tests were the elastic modulus, E, as the slope of the curve between 0% and 2% of elongation, the tensile strength, and the elongation at the yield (σ_y_ and ε_y_) and at break points (σ_b_ and ε_b_), each one of them reported as the average value from each test. Nevertheless, since the Box–Wilson methodology does not contemplate the processing step but only a series of independent terms, the results obtained will be related to the interactions between components. In particular, in our system, the variables that can influence the final properties will be the amount of the components of the electrospun nanocomposites (PLA, MgO, and OLA). We used the same dispersion and electrospinning process parameters for all the experimental runs in order to reasonably control its effect in the final mechanical performance of the electrospun nanocomposites.

## 3. Results and Discussion

**Figure 1:**
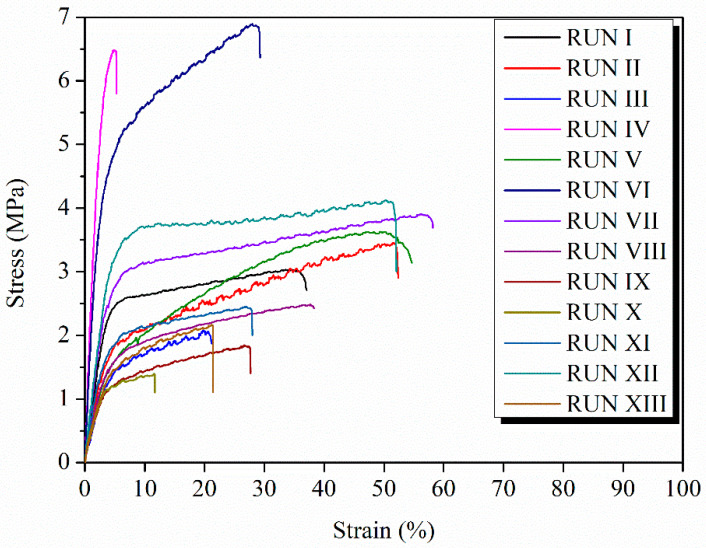
Stress–strain curve for PLA-based efibers obtained in each run studied.

Once the different woven non-woven electrospun nanocomposites mats were obtained, the mechanical characterization of each run was carried out. Table 2 compiles all the results obtained from the tensile test experiments according to the Box–Wilson two independent variables experimental worksheet—in particular, the mechanical properties measured in the main regions at the stress–strain curves: the elastic zone (elastic modulus), and the yield and break points (strength and elongation in both). In Figure 1, the stress–strain curves for each run of the PLA-based efibers is reported.

As can be observed, the stress–strain curves are widely affected by the different compositions of each experimental run according to the Box–Wilson two independent variables experimental worksheet (Table 1). However, the central points—that is, from run IX to run XIII—showed similar tensile behaviors due to their similar compositions in terms of both OLA (wt%) and MgO (wt%) contents.

The results obtained for each one of the studied parameters were fitted to quadratic models by following the Box–Wilson surface response methodology [39]. Five different polynomials describing the evolution of E (MPa), σ_y_ (MPa), ε_y_ (%), σ_b_ (MPa), and ε_b_ (%) were properly obtained, having the general form

Y = a_0_ + a_1_∙x_1_ + a_2_∙x_2_ + a_3_∙x_1_∙x_2_ + a_4_∙x_1_^2^ + a_5_ x_2_^2^

The coefficients obtained for each polynomial fit are summarized in Table 3, as well as its percentual confidence values for <r^2^> (%), the lack of fit, and the confidence factors coefficients, obtained from the ANOVA.

As reported in the literature [39,40], <r^2^> (%) is the statistical parameter that shows the quality of the fit of the model to a dataset and its predictive capability for the studied variables. In addition, <r^2^> (%) values higher than 70.00% are considered indicative of good fitted quadratic models. In our case, the <r^2^> (%) values obtained were 82.47% (E), 73.52% (σ_y_), 69.12% (ε_y_), 67.29% (σ_b_), and 45.79% (ε_b_), respectively. Therefore, in our case, for E and σ_y,_ we can remark on the significance of the independent variables chosen, which are, in this case, the contents of both OLA (wt%) and MgO (wt%), to model the mechanical behavior of the PLA-based efibers in the experimental range studied. On the other hand, the <r^2^> (%) values for ε at yield and σ at break, 69.12% and 67.29% respectively, are in the limit and particularly low for ε at yield (45.79%) due to the wide variability of the experimental elongation values at the break point.

Table 3 also shows the lack of fit values, which are the percentage of pure error, related to possible factors not considered by the model but significantly influencing the response. High values of lack of fit suggest that this parameter is more susceptible to the noise effects present in the experiment process. Additionally, high values for the confident factor indicate the full significance of the independent variables chosen in the study. As described above, in our case, the OLA (wt%) and MgO (wt%) contents properly described the mechanical behavior of efibers. In particular, lack of fit values for the elastic modulus, 51.0%, and tensile strength at break, 62.2%, are the highest, which indicates that these are the parameters in which pure error is easier to accumulate. This is in agreement with the fact that both of them can be considered as the most sensitive parameters to the stress transmission between the PLA matrix and the MgO NPs, being, respectively, the starting and the end points of the mechanical test.

Likewise, very high values of the “confidence factor” indicate that almost all the factors considered to build the model play a prime role in the mechanical behavior of the system, except the elongation at break. As described above, this parameter showed a wide variability of the experimental values measured, leading to the lowest confidence factor value of 59.1%.

**Figure 2:**
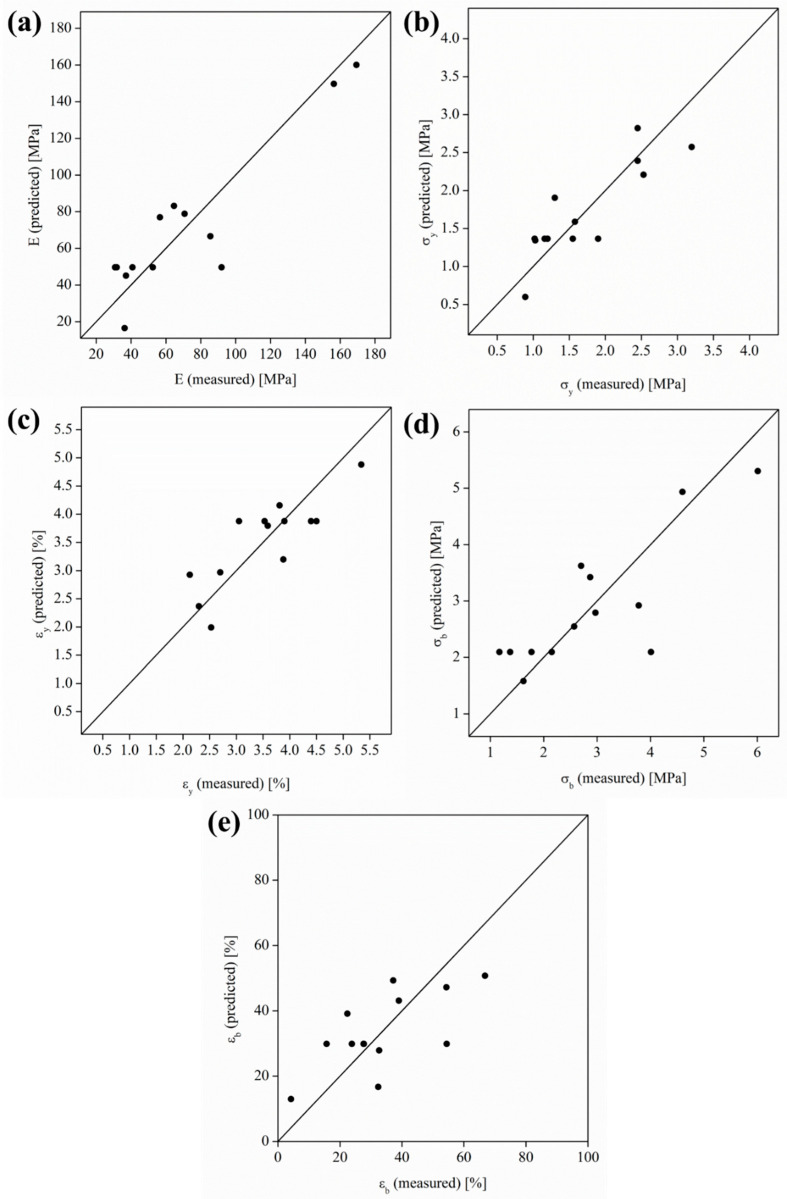
Predicted versus measured values for (**a**) E, (**b**) σ_y_, (**c**) ε_y_, (**d**) σ_b_, and (**e**) ε_b_.

Additionally, in order to check the limitations of the model, Figure 2 shows the predicted versus the measured properties for the different mechanical properties. A good correlation between the predicted and measured values is observed but with some variability. As can be seen, homoscedastic distribution of the predicted values with respect to the measured one can be observed for all the mechanical properties studied.

Otherwise, Table 4 shows the confidence coefficient (%) and *t*-value related to the different terms of the Box–Wilson model obtained for the studied properties. As described in the literature, *t*-values higher than 2 are considered statistically significant, and the higher the *t*-value for a term, the greater the confidence in that particular term [39,40]. Among all the studied mechanical properties, E and σ at yield are the only ones able to show significant *t*-values higher than 2 for the linear terms of each independent variable, OLA (wt%) and MgO (wt%), as well as for the interaction term. Additionally, the elastic modulus showed a significant *t*-value of 2.54 for the quadratic term related to the OLA (wt%) content, which indicated the complexity of the interaction between the PLA matrix and the plasticizer. For the mechanical properties measured at the break point—that is, σ_b_ and ε_b_—some considerations can be done. In particular, σ_b_ showed significant *t*-values higher than 2 for the linear and the quadratic terms related to OLA (wt%), while ε_b_ showed significant *t*-values higher than 2 only for the independent terms, which is in agreement with the previously described variability of the experimental elongation values measured at the break point, suggesting that, for this specific mechanical property, ε_b_, the choice of both the OLA and MgO contents as independent variables is not the more adequate one.

### 3.1. Influence of the OLA and MgO Contents in the Elastic Modulus of Efibers

**Figure 3:**
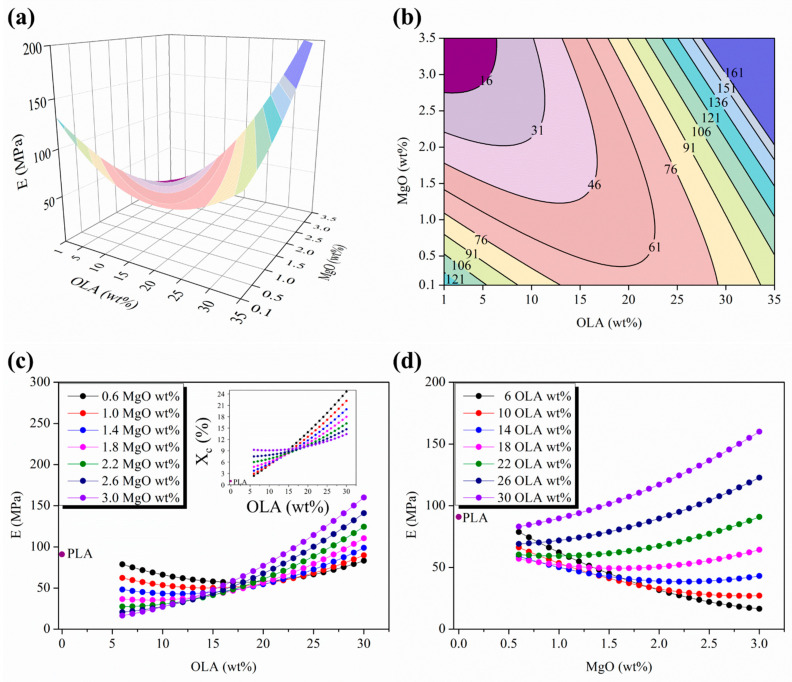
(**a**) Three-dimensional response surface plot and (**b**) contour plot of the elastic modulus as a function of the OLA and MgO NPs contents; colors changes are attributed to an increment of 15 MPa in the elastic modulus of efibers. (**c**) Parametric evolution of the elastic modulus with the OLA content remaining constant at the MgO NP level. (**d**) Parametric evolution of the elastic modulus with the MgO NP contents remaining constant at the OLA amounts.

Figure 3a shows the 3D response surface plot and Figure 3b the isolines map of the elastic modulus, E, as a function of the OLA (wt%) and MgO (wt%) contents. It is shown as a convex saddle-shaped function not centered in the experimental space scanned and well defined in the region around the 15–22 wt% amount of OLA and above the 1 wt% amount of MgO.

In order to identify the critical points, Figure 3c,d show the parametric evolutions of the elastic modulus, E, with the OLA (wt%) and the MgO (wt%) contents, respectively, while the other one remains constant. In addition, the E values of neat PLA efibers have been included in these plots in order to compare with those of the different PLA-based efibers (purple dots in Figure 3c,d). It is important to remark that only the PLA-based efibers with OLA contents above 15 wt% and MgO contents above 1.4 wt% approach or overpass the E value of neat PLA efibers (E = 91 ± 8 MPa [17]). In fact, we can define a critical point at 15 wt% of OLA and below 15 wt% of OLA; all the PLA-based efibers show E values lower than the E value of neat PLA efibers and increase when the amount of MgO (wt%) decreases. On the other hand, above 15 wt% OLA, the PLA-based efibers with the lowest contents of MgO (lower than 1.4 wt% MgO) showed E values lower than neat electrospun PLA. However, once the amount of MgO (wt%) reaches and passes the 1 wt% content of MgO, the PLA-based efibers show the higher E values, overpassing the E value of neat PLA efibers. In particular, from 2 wt% MgO, the highest E values were observed.

Therefore, it is important to point out that the minimum in the evolution of E values takes place at 15 wt% of OLA, in agreement with the convergence point described in the evolution of the degree of crystallinity, X_c_, values widely discussed in our previous work [18]. In order to compare the region that identifies the minimum stationary points placed at the surface responses of the electrospun PLA-based nanocomposites, both in terms of E as well as of X_c_, an insert of the parametric evolutions of X_c_ with the OLA (wt%) content while the MgO (wt%) content remains constant has been included in Figure 3c.

As can be seen in Figure 3c, the highest E values, as well as the highest X_c_ values, are observed at the similar OLA (wt%) content regions—that is, above 15 wt%—but with inverse behavior related to the content of MgO (wt%). For instance, at the highest amount of OLA, 30 wt%, the highest E values were observed for the highest amount of MgO, 3 wt%, while, for the same amounts of OLA (wt%) and MgO (wt%), the X_c_ values was the lowest observed (purple isolines in Figure 3c).

The inversion between the E and the X_c_ evolutions on each corresponding parametric MgO (wt%) plots was discussed in a previous work [18] and, in this work, will be correlated with the mechanical response of the nanocomposites.

In fact, the decrease in the E values of the PLA-based nanocomposites with respect to the E values of neat PLA efibers and increasing the X_c_ (%) values always higher than the X_c_ (%) values of neat PLA efibers confirm the poor contribution of the PLA matrix to the stiffness of PLA-based nanocomposites. In terms of mechanical behavior, it means that PLA evolves as an almost amorphous polymeric material, which otherwise agrees with the T_g_ evolution of these PLA-based nanocomposites, as previously discussed [18].

From this perspective, the parametric evolutions of the elastic modulus, E, with the MgO (wt%) content for different contents of OLA (wt%) displayed in Figure 3d allows to identify 1.5 wt% as the critical amount of MgO; above which, the plasticizing effect of OLA that tends to decrease the E values is weaker as the amount of both OLA (wt%) and MgO (wt%) increase, until completely disappearing above the 15 wt% amount of OLA, where PLA-based efibers give the highest E values, even overcoming the E values of neat PLA efibers.

### 3.2. Mechanical Behavior at the Yield Point of the Woven Non-Woven PLA-Based Efibers

**Figure 4:**
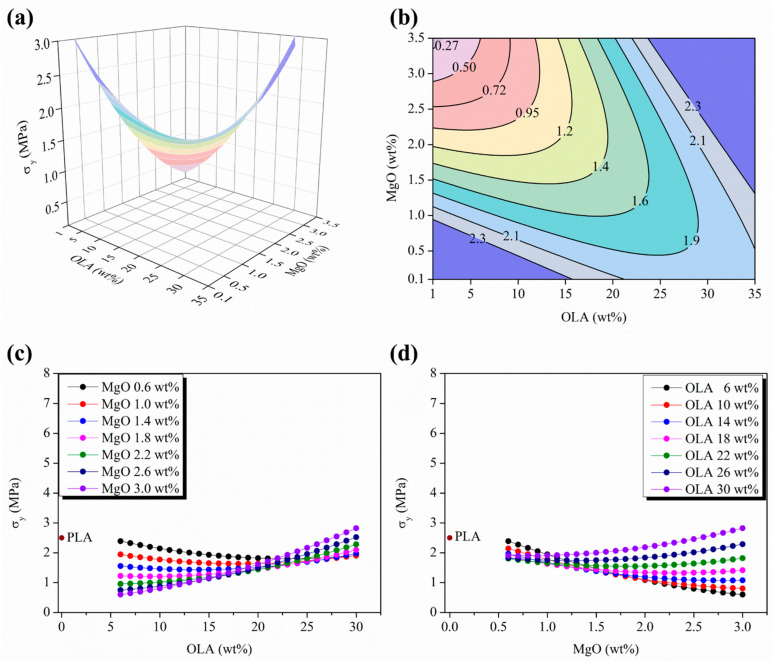
(**a**) Three-dimensional response surface plot and (**b**) contour plot of the σ_y_ as a function of OLA and MgO NPs contents; color changes are attributed to an increment of 0.2 MPa in the σ_y_ of efibers. (**c**) Parametric evolution of σ_y_ with the OLA content keeping constant the MgO NP levels. (**d**) Parametric evolution of σ_y_ with the MgO NPs content keeping constant the OLA amounts.

**Figure 5:**
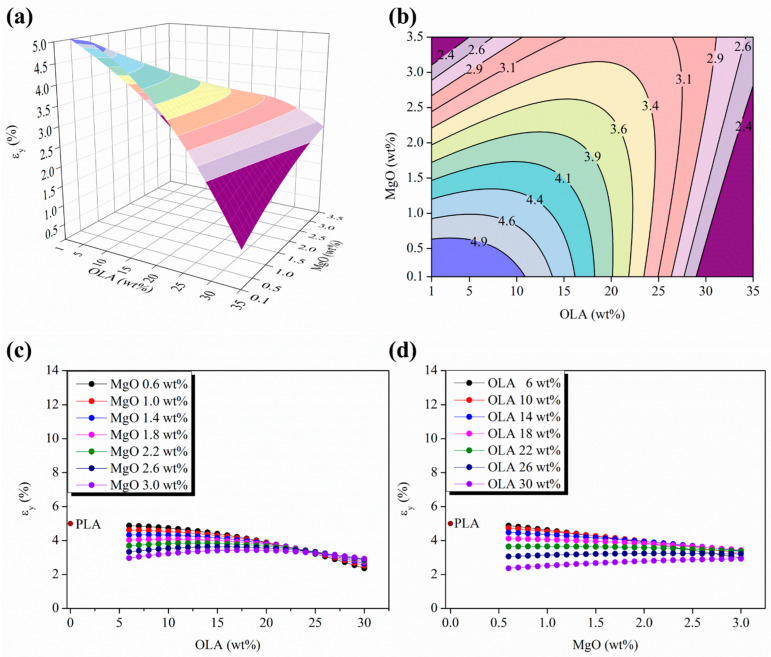
(**a**) Three=dimensional response surface plot and (**b**) contour plot of the ε_y_ as a function of the OLA and MgO NPs content; color changes are attributed to an increment of 0.3% in the ε_y_ of efibers. (**c**) Parametric evolution of ε_y_ with the OLA content keeping constant the MgO NPs level. (**d**) Parametric evolution of ε_y_ with the MgO NPs content keeping constant the OLA amounts.

Figure 4 and Figure 5 show the stress and strain responses for the woven non-woven PLA-based efibers at the yield point, respectively. As can be seen, the expected behavior for a tensile test carried out at room temperature is shown. Indeed, from the early stages of the strain processes under normal stress conditions, the matrix polymer chains tend to minimize the external force effects by flowing and then orienting parallel to the applied load direction. Then, for neat PLA efibers, the yield point indicates where the polymer chains of the PLA matrix are able to drag between them, continuing the strain of the material while the stress level remains constant. However, for PLA-based efibers, the other components such as the OLA chains and MgO NPs, would be unable to participate in the drag mechanism—the OLA chains because of their short lengths and the MgO NPs due to their stiffness. In both cases, the loss of interactions at nanoscale level between the NPs and the surrounding polymeric chains in those regions under strain processing at constant stress is expected, which provokes a decrease in the yield point values with respect to those of neat PLA efibers.

Accordingly, the stress reached at the yield point, defined as the yield strength, σ_y_, is identified as the point where the slope on a stress–strain curve becomes zero. Figure 4a,b show the 3D response surface plot and the isolines map of the σ_y_, respectively, in the experimental space scanned as a function of both the OLA (wt%) and the MgO (wt%) contents, respectively.

As previously discussed for the evolution of the elastic modulus, E, the σ_y_ response surface evolves as the convex side of a saddle-shaped function, with the minimum stationary points placed almost symmetrical in both sides of the experimental space scanned, as observed in the isolines plots in Figure 4b. The minimum distances between isolines appear at the lowest and the highest limits of the experimental space scanned for the OLA (wt%) and the MgO (wt%) contents, respectively, which allow to identify the higher sensitivity σ_y_ response regions at the minimum variations in the amounts of OLA (wt%) and MgO (wt%).

Furthermore, the parametric plots shown in Figure 4c,d include the σ_y_ value of neat woven non-woven PLA efibers, 2.6 MPa. Only the PLA-based nanocomposites with a content of OLA of 30 wt% and a content of MgO higher than 2.6 wt%, respectively, exceed this value. To note again the excellent agreement with the elastic modulus evolution, it is important to remark that the convergence point for the σ_y_ curves is placed above 20–25 wt% OLA and slightly above E evolution 15 wt%. In particular, below this point, OLA 20–25 wt%, the σ_y_ values slightly decrease for the lowest MgO (wt%) content by increasing the OLA (wt%) content, remaining always below the σ_y_ value for PLA efibers. From the convergence point at OLA 20–25 wt%, increasing the MgO content up to 2.2 wt% allows to overpass the σ_y_ value of neat PLAs, just as it was found for the E evolution in Figure 3c. Above the convergence point in σ_y_ evolution, better interactions between NPs and the PLA matrix due to the presence of OLA was evidenced by the fact that the σ_y_ increased as the MgO content increased from 2.2 to 3.0 wt%. On the other hand, below the 2.2 wt% MgO content, the σ_y_ evolution isolines almost overlapping until the lowest MgO content, 0.6 wt%.

Moreover, the response surface and isoline maps of the strain evolution at the yield point, ε_y_ (%), as a function of the OLA (wt%) and MgO (wt%) contents is shown in Figure 5a,b.

In this case, the response surface evolves as a saddle-shaped function inverting its curvature with respect to those previously discussed for both the E and the σ_y_ evolution. As can be seen in the isolines map in Figure 5d, the isoline distances decrease in the limits of the experimental space scanned for the OLA (wt%) and the MgO (wt%) contents, which is in total agreement with the previously described results for the E and the σ_y_ response surfaces.

In order to compare the evolution of the elongation at yield for the different PLA-based efibers, the parametric plots in Figure 5c,d show the ε_y_ values for the neat PLA efibers. In Figure 5c, the strain at yield values always decrease in the PLA-based efibers with respect to the PLA matrix, and tend to converge in a wide range of OLA contents between 20 and 25 wt%, which agrees with the previous results describing the σ_y_ evolution in Figure 4c. Otherwise, Figure 5d shows an almost lineal decrease of ε_y_ as the MgO (wt%) content increases, with smoothing and decreasing slopes up to zero for the OLA 22 wt% content. From this amount of OLA, 22 wt% (green isoline in Figure 5d), the ε_y_ isolines raised a constant value of ε_y_ in the experimental space scanned for MgO (wt%). However, once overpassing the OLA 22 wt% content, the ε_y_ values show slightly positive slopes for OLA contents of 26 and 30 wt%, respectively (dark blue and purple isolines in Figure 5d).

From the mechanical point of view at the yield point, in our PLA-based efibers, the presence of OLA in a range of concentration from 20 up to 30 wt% leads the system to approach a slight improvement in the yielding capability in terms of σ_y_ in comparison with neat woven non-woven PLA efibers. In the range of concentration of OLA between 20 and 30 wt%, the highest σ_y_ values were observed, which can be attributed to an improvement in the interaction level between the surrounding PLA polymeric chains and the NPs.

### 3.3. Influence of OLA and MgO Contents in the Mechanical Behavior at the Break Point of Efibers

**Figure 6:**
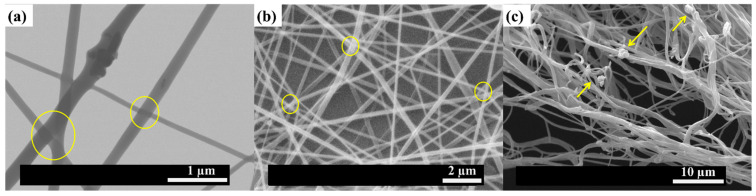
(**a**) FESEM images of PLA-based efibers from run X. (**b**) SEM images of tensile specimens of PLA-based efibers from run X before the tensile test, and (**c**) fracture surfaces of the same tensile specimens of PLA-based efibers from run X after the tensile test. Yellow circles are attributed to points where randomly oriented efibers contacted between them during the electrospinning process.

The final breakage of PLA-based efibers is observed at the end of the tensile tests. This breakage, will occur at the mechanically weakest points of the highly strained system, which could be the points where randomly oriented efibers contacted between them during the electrospinning process (yellow circles in Figure 6a,b). However, other weak points would be those points close to the shortest polymer chains involved in the drag mechanism or the points on the surfaces of NPs embedded in PLA chains undergoing the strain process.

**Figure 7:**
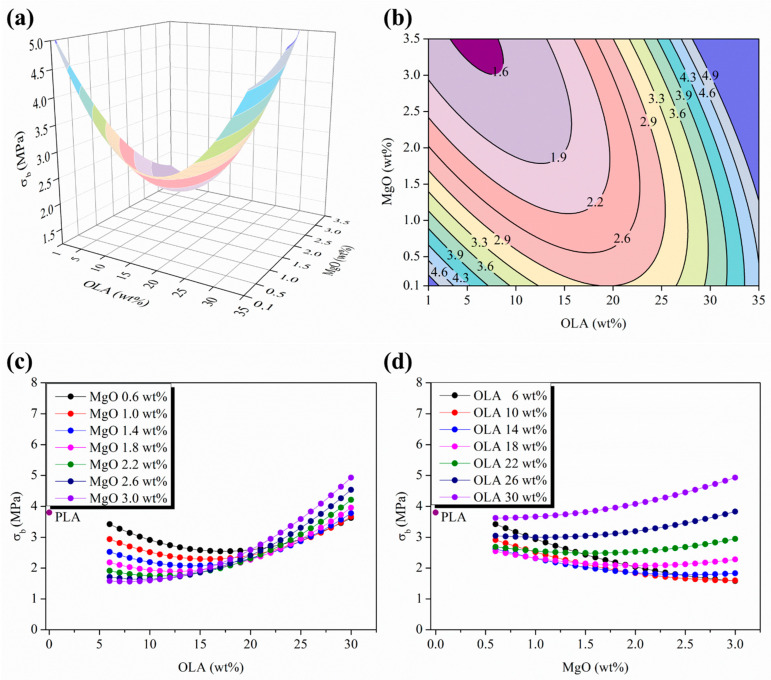
(**a**) Three-dimensional response surface plot and (**b**) contour plot of the σ_b_ as a function of the OLA and MgO NPs contents; color changes are attributed to an increment of 0.4 MPa in the σ_b_ of efibers. (**c**) Parametric evolution of σ_b_ with the OLA content keeping constant the MgO NPs level. (**d**) Parametric evolution of σ_b_ with the MgO NPs content keeping constant the OLA amounts.

In order to verify the previous proposed tensile mechanism, the break point evolution of the woven non-woven efibers was studied in terms of σ_b_ (MPa) and ε_b_ (%). First of all, Figure 7a,b show both the 3D response surface plot and the isolines map of the σ_b_ evolution as a function of the OLA (wt%) and MgO (wt%) contents.

From the σ_b_ evolution point of view, in Figure 7b is observed a convex response surface well characterized by the presence of a minimum stationary point outside the experimental plane scanned moving towards the highest amount of MgO (wt%) and the lowest amount of OLA (wt%). As can be seen in Figure 7c,d, the σ_b_ values of PLA-based efibers are always lower than those of neat PLA efibers; only the electrospun nanocomposites with the highest content of OLA 30 wt% overcome the σ_b_ value of neat PLA when the content of MgO overpasses 1.5 wt%. As previously observed for the mechanical properties at the yield point, a convergence point located at OLA 20 wt% was observed, from which, by increasing the MgO (wt%) content yields, increases the σ_b_ values for the PLA-based efibers, which is in agreement with the previously described evolution for the elastic modulus, as well as the tensile strength at yield.

**Figure 8:**
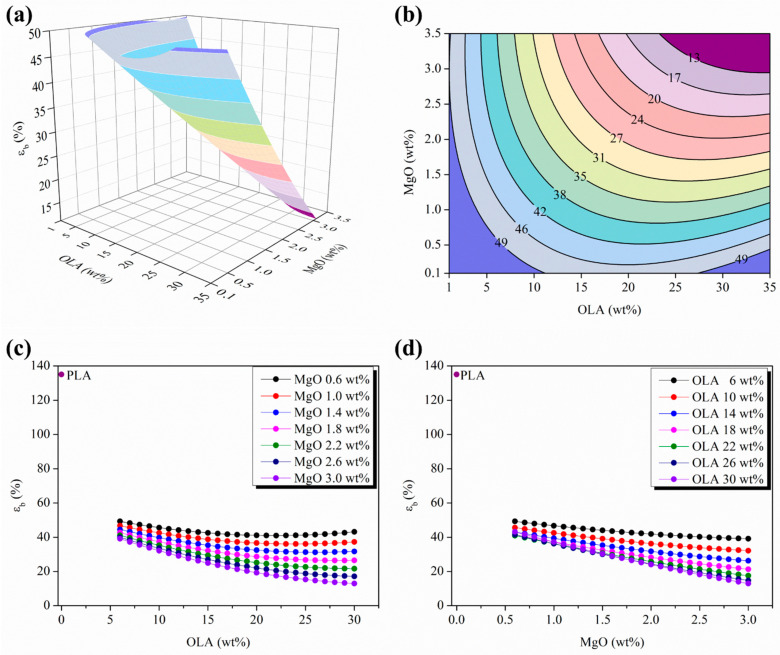
(**a**) Three-dimensional response surface plot and (**b**) contour plot of the ε_b_ as a function of the OLA and MgO NPs contents; color changes are attributed to an increment of 4% in the ε_b_ of efibers. (**c**) Parametric evolution of ε_b_ with the OLA content keeping constant the MgO NPs level. (**d**) Parametric evolution of ε_b_ with the MgO NPs content keeping constant the OLA amounts.

The described results obtained for the σ_b_ are correlated with the evolution of ε_b_ for the woven non-woven PLA-based efibers. Therefore, Figure 8 shows both the 3D response surface plot and the isolines map evolution of the ε_b_ as a function of the OLA (wt%) and the MgO (wt%) contents. In Figure 8a,b are observed a convex response surface with a very low decreasing slope moving towards the limits of the experimental space scanned for both the OLA (wt%) and the MgO (wt%) contents. Moreover, in Figure 8b, the isolines distance remains almost constant all along the contour map, suggesting an almost ε_b_ linear evolution in good agreement with the breakage of a highly strained material once it ended its yield capabilities.

In contrast with the previously discussed results for other mechanical properties, as it can be seen from the parametric plots shown in Figure 8c,d, the ε_b_ values are about 3 to 14 times lower than the ε_b_ values for neat PLA efibers in the experimental space scanned. Moreover, it is important to remark on the disappearance of the convergence point previously observed at OLA 20 wt%. In addition, in Figure 8c,d, both the parametric evolution of the ε_b_ versus OLA (wt%) and MgO (wt%) contents, respectively, showed almost linear and negative slopes of ε_b_ evolution as the contents of OLA (wt%) or MgO (wt%) increased, which is in excellent agreement with the previously proposed tensile mechanism.

In order to support the previously discussed tensile mechanism of the woven non-woven PLA-based efibers, SEM and FESEM images of PLA-based efibers are shown in Figure 6. These images correspond to a specimen of PLA-based efibers of the central point of the Box–Wilson worksheet—in particular, run X (18 wt% OLA and 1.80 wt% MgO). As previously discussed, these amounts of OLA (wt%) and MgO (wt%) are in the region of the critical point where the NPs are able to participate in the overall stress transmission mechanism in an efficient manner.

First of all, some considerations have to be taken into account. Electrospun woven non-woven mats are a kind of material constituted by fibers with homogeneous morphology and free spaces between them [35]. In addition, the framework of efibers usually shows some contact points between fibers (yellow circles), as observed in Figure 6a,b.

**Figure 9:**
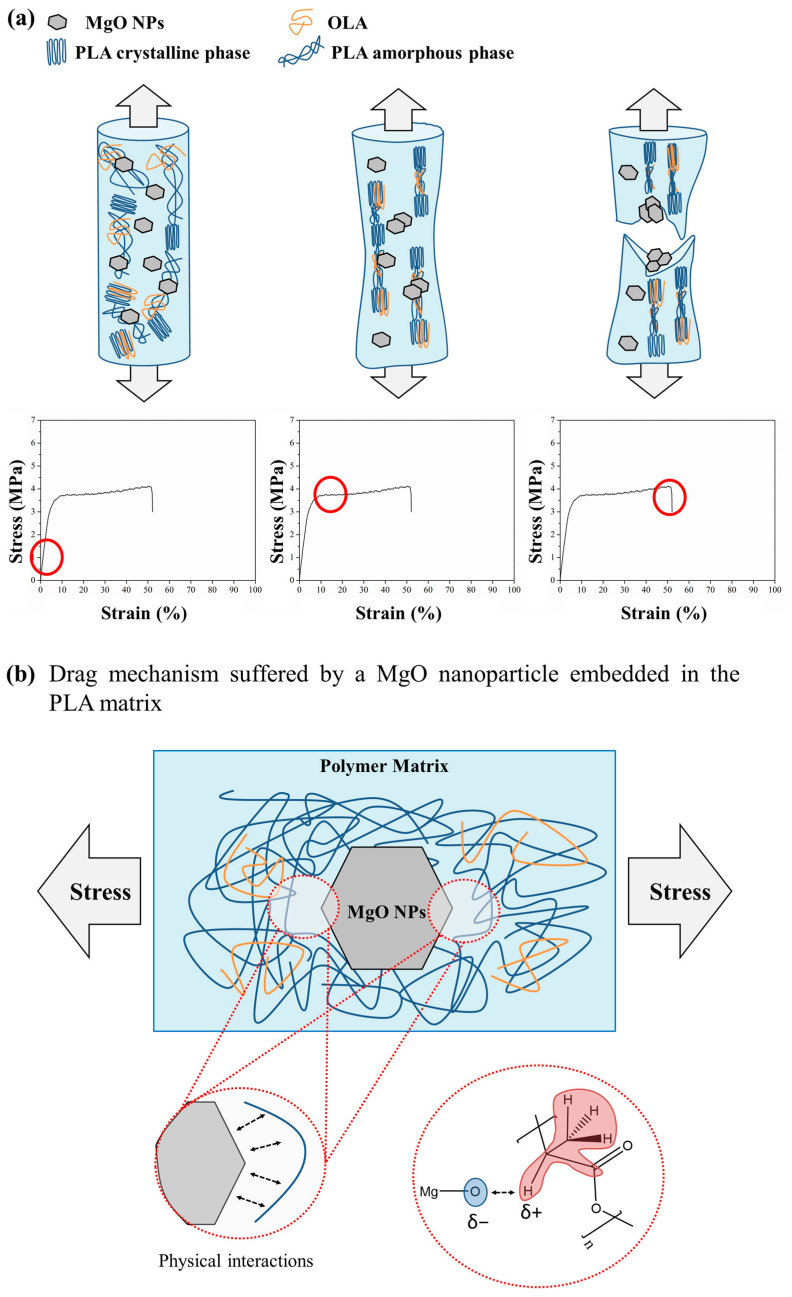
(**a**) Schematic representation of the mechanical behavior of the PLA-based efibers during the different stages of the tensile test. Red circles are attributed to the elastic region, yield point and break point (**b**) Proposed strain mechanism of PLA/OLA/MgO efibers.

Due to the structure of electrospun woven non-woven mats, it is impossible to observe the whole tensile fracture surface of continuous materials. Thus, when electrospun woven non-woven mats are exposed to an external stress until the break point is reached, each efiber in the woven non-woven mat will break individually. As previously proposed, once past the yield point, polymer chains will start to align parallel to the applied force direction, and therefore, the embedded NPs will be dragged by them. Thus, the MgO NPs dispersed in the PLA matrix may be considered as stress concentrators where the breakage of the materials will start. It can be assumed that, accordingly with those previously exposed, the fractures will initiate around the stress concentration sites and lead to the final breakage of the fibers, as is schematically represented in Figure 9. Moreover, this behavior is similarly described in the literature [41]. For instance, Curgul et al. reported how the overall deformation of a fiber is determined by mobility of the polymer chains at the surface of the fiber and the number of oriented fragments present once past the yield point [42]. In addition, Arienstein et al. concluded that the orientation of the amorphous chains in the supramolecular region of the fibers influences the mechanical deformation process of the fibers [43]. On the other hand, Kim et al. proposed a similar model representation for the mechanical deformation process of electrospun polymer nanocomposite fibers in good agreement with our proposed mechanism shown in Figure 9a. In particular, they described how the presence of nanofillers within the fibers caused the stress concentration and acted as effective stress concentrators under the tensile load, because they have quite different elastic properties from those of the polymer matrix [44].

In order to support the described strain mechanism, SEM images both before and after the tensile test are reported in Figure 8. Firstly, Figure 8a shows the MgO NPs embedded through the efibers, confirming the good dispersion of NPs by the electrospinning process carried out. Moreover, randomly oriented and smooth efibers can be observed in Figure 8b before the tensile test. After carrying out the tensile test, the fracture surface of the specimen was observed by SEM and is shown in Figure 8c. Firstly, the efibers showed an orientation parallel to the applied stress. In addition, as can be seen and it is indicated by the arrows, the broken efibers showed MgO NPs in the broken surface, corroborating the tensile mechanism. Once the PLA-based efibers are highly strained, the surfaces of MgO NPs in contact with PLA chains are the mechanically weakest points, yielding to the breakage of the efibers at these points.

## 4. Conclusions

The mechanical behaviors of woven non-woven PLA/OLA/MgO efibers were determined by studying their mechanical properties measured in the main regions at the stress–strain curves: the elastic zone (elastic modulus) and the yield and break points (strength and elongation in both). A Box–Wilson model was used in order to identify the level of interactions and look for the optimal compositional ratios. In our case, the <r^2^> (%) values obtained were 82.47% (E), 73.52% (σ_y_), 69.12% (ε_y_), 67.29% (σ_b_), and 45.79% (ε_b_), respectively. In addition, the predicted versus the measured values for E, σ_y_, ε_y_, σ_b_, and ε_b_ showed very good correlations, evolving as homoscedastic distributions.

The parametric evolutions of the elastic modulus, E, with the MgO (wt%) contents for different contents of OLA (wt%) allowed to identify 1.5 wt% as the critical amount of MgO; above that, the plasticizing effect of OLA is weaker as the amount of both OLA (wt%) and MgO (wt%) increase. Moreover, the minimum E values take place at 15 wt% of OLA, in agreement with the previously reported convergence point in the evolution of the degree of crystallinity, X_c._

On the other hand, from the mechanical point of view, at the yield point, the presence of OLA in a range of concentrations from 20 to 30 wt% leads the system to approach a slight improvement in the yielding capability in terms of σ_y_ in comparison with neat woven non-woven PLA efibers. In the range of concentrations of OLA between 20 and 30 wt%, the highest σ_y_ values were observed, which could be attributed to an improvement in the interaction level between the surrounding PLA polymeric chains and the NPs.

Moreover, the strain mechanism of PLA/OLA/MgO efibers was described. Firstly, the efibers showed an orientation parallel to the applied stress. Then, once the material reached the yield point, polymer chains started to align parallel to the applied force direction, and therefore, the embedded NPs were dragged by them. Finally, when the PLA-based efibers were highly strained, the surfaces of the MgO NPs in contact with PLA chains were the mechanically weakest points, yielding to the breakage of the efibers at these points.

## Figures and Tables

**Table 1:** Worksheet for the Box–Wilson experimental design used.

	Coded Factors		Controlled Factors	
Run	OLA	MgO	OLA (wt%)	MgO (wt%)
I	−1	−1	6.00	0.60
II	1	−1	30.00	0.60
III	−1	1	6.00	3.00
IV	1	1	30.00	3.00
V	−√2	0	1.03	1.80
VI	√2	0	34.97	1.80
VII	0	−√2	18.00	0.10
VIII	0	√2	18.00	3.49
IX	0	0	18.00	1.80
X	0	0	18.00	1.80
XI	0	0	18.00	1.80
XII	0	0	18.00	1.80
XIII	0	0	18.00	1.80

**Table 2:** Average mechanical properties obtained from the tensile test of each run.

Run	E (MPa)	σ_y_ (MPa)	ε_y_ (%)	σ_b_ (MPa)	ε_b_ (%)
I	70.8 ± 9.5	2.5 ± 0.8	5.3 ± 0.6	2.9 ± 1.1	37.2 ± 1.6
II	64.7 ± 8.9	1.3 ± 0.7	2.3 ± 0.2	2.7 ± 1.8	39.0 ± 1.3
III	36.3 ± 4.5	0.9 ± 0.3	2.7 ± 0.6	1.6 ± 0.4	22.4 ± 2.1
IV	169.4 ± 6.0	2.5 ± 0.6	2.1 ± 0.6	4.6 ± 1.8	4.2 ± 1.3
V	37.1 ± 7.9	1.0 ± 0.3	3.6 ± 0.4	2.9 ± 0.7	66.8 ± 1.7
VI	156.4 ± 9.3	3.2 ± 0.5	2.5 ± 0.4	6.0 ± 1.2	32.7 ± 1.7
VII	85.6 ± 2.7	2.5 ± 0.5	3.8 ± 0.7	3.8 ± 0.8	54.3 ± 1.4
VIII	56.6 ± 1.3	1.6 ± 0.4	3.9 ± 0.6	2.6 ± 0.6	32.4 ± 1.2
IX	40.9 ± 1.7	1.0 ± 0.4	3.1 ± 0.2	1.8 ± 0.4	27.6 ± 1.5
X	30.8 ± 1.8	1.2 ± 0.3	3.5 ± 0.1	1.2 ± 0.6	15.7 ± 1.7
XI	52.5 ± 9.9	1.6 ± 0.3	3.9 ± 0.5	2.2 ± 0.4	27.7 ± 1.7
XII	91.9 ± 2.4	1.9 ± 0.2	4.5 ± 0.5	4.0 ± 0.8	54.4 ± 1.4
XIII	31.8± 3.1	1.2 ± 0.4	4.4 ± 0.7	1.4 ± 0.8	23.8 ± 1.7

**Table 3:** Statistical parameters and coefficients of the polynomial equations from the Box–Wilson experimental design used (Y = a_0_ + a_1_∙x_1_ + a_2_∙x_2_ + a_3_∙x_1_∙x_2_ + a_4_∙x_1_^2^ + a_5_∙x_2_^2^).

	<r^2^> (%)	L. F. * (%)	C. F. * (%)	Ind. T. *	L. T. *		Int. T. *	Q. T. *	
				a_0_	a_1_	a_2_	a_3_	a_4_	a_5_
E (MPa)	82.47	51.0	97.8	145.7	−7.248	−68.16	2.416	0.1661	7.699
σ_y_ (MPa)	73.52	16.1	93.9	3.833	−0.1225	−1.695	0.04705	0.002058	0.1850
ε_y_ (%)	69.12	31.4	90.8	5.402	−0.007468	−0.8061	0.04288	−0.003415	−0.06892
σ_b_ (MPa)	67.29	62.2	89.3	5.648	−0.2686	−1.893	0.05469	0.006783	0.2217
ε_b_ (%)	45.79	32.2	59.1	59.91	−1.228	−5.980	−0.3477	0.03279	1.067

* L. F. (lack of fit), C. F. (confident factor), Ind. T. (independent term), L. T. (linear terms), Int. T (interaction term), and Q. T. (quadratic terms).

**Table 4:** Confidence coefficients (%) and *t*-values for the different terms of the Box–Wilson model obtained for the mechanical properties (Polynomial Equation: a_0_ + a_1_∙x_1_ + a_2_∙x_2_ + a_3_∙x_1_∙x_2_ + a_4_∙x_1_^2^ + a_5_∙x_2_^2^).

	Ind. T. *	L. T. *		Int. T. *	Q. T. *	
		x_1_	x_2_	x_1_·x_2_	x_1_^2^	x_2_^2^
E (MPa)	3.52 (98.9%)	2.49 (95.9%)	2.34 (95.0%)	2.80 (97.4%)	2.54 (96.2%)	1.18 (71.0%)
σ_y_ (MPa)	4.68 (99.6%)	2.12 (93.0%)	2.94 (97.8%)	2.75 (97.2%)	1.59 (84.1%)	1.43 (79.7%)
ε_y_ (%)	4.72 (99.6%)	0.09 (19.1%)	1.00 (63.3%)	1.78 (88.4%)	1.89 (90.0%)	0.38 (31.7%)
σ_b_ (MPa)	3.23 (98.5%)	2.18 (93.6%)	1.54 (82.8%)	1.50 (81.9%)	2.46 (95.8%)	0.80 (53.6%)
ε_b_ (%)	2.19 (93.7%)	0.64 (44.9%)	0.31 (28.3%)	0.61 (43.5%)	0.76 (51.2%)	0.25 (25.4%)

* Ind. T. (independent term), L. T. (linear terms), Int. T (interaction term), and Q. T. (quadratic terms).

## Data Availability

Not applicable.
